# Complement activation contributes to GAD antibody-associated encephalitis

**DOI:** 10.1007/s00401-022-02448-x

**Published:** 2022-06-13

**Authors:** Omar Chuquisana, Christine Strippel, Anna M. Tröscher, Tobias Baumgartner, Attila Rácz, Christian W. Keller, Christian E. Elger, Nico Melzer, Stjepana Kovac, Heinz Wiendl, Jan Bauer, Jan D. Lünemann

**Affiliations:** 1 Department of Neurology With Institute of Translational Neurology, University Hospital Münster, 48149 Münster, Germany; 2 Department of Neuroimmunology, Center for Brain Research, Medical University of Vienna, Vienna, Austria; 3 Department of Neurology I, Neuromed Campus, Kepler University Hospital Linz, Linz, Austria; 4 Department of Epileptology, University Hospital Bonn, Bonn, Germany; 5 Department of Neurology, Medical Faculty, Heinrich-Heine-University Düsseldorf, Düsseldorf, Germany

Antibody (Ab)-mediated autoimmune encephalitides (AE) comprise a group of rare and severe inflammatory brain diseases, which share key biological and clinical features, such as prominent neuropsychiatric symptoms, occurrence of Abs specific for neuronal cell antigens (Ags), and frequent residual deficits [2]. While Abs targeting neuronal cell surface Ags (NSAb) are pathogenic, as it has been demonstrated in vitro and in animal models of passive and active immunization [1, 3–5], AE with Abs specific for intracellular proteins such as the 65 kDa isoform of glutamic acid decarboxylase (GAD65) are thought to be mediated by T cells [1].

The mechanisms by which AE-related Abs mediate pathology and how Ab-mediated effector functions translate into clinical syndromes are poorly understood. Pathogenic mechanism mediated by the Ag-binding domain (Fab) of IgG Abs includes internalization of targeted cell surface proteins or allosteric modulation of protein functioning, affecting neural signaling. Pathogenic effector functions mediated by the crystallizable domain (Fc) potentially include recruitment and activation of immune cells through engagement of activating Fc-γ receptors and activation of the complement cascade.

Here, we systemically profiled complement activation in immunotherapy-naive patients with neuronal cell surface-specific Abs, patients with GAD-Ab^+^ encephalitis, patients with relapsing–remitting multiple sclerosis (RMS) and healthy subjects (HD) (Supplementary Table 1).

Compared to people with RMS, patients with the diagnosis of AE showed substantially elevated CSF levels of activated complement proteins (Fig. 1a). Increased CSF levels were observed in patients with GAD-Ab^+^ encephalitis and patients with NSAbs. The variability observed in CSF activated protein concentration might partly result from Ab binding to the two GAD isoforms, GAD65 and GAD67. In contrast to the CSF compartment, serum levels complement proteins were unchanged in patients with AE compared to patients with RMS and HD with the exception of C4a (Fig. 1a). Thus, patients with GAD Ab^+^ and NSAb-associated AE showed increased levels of activated complement proteins within the CSF compartment.

**Fig. 1:**
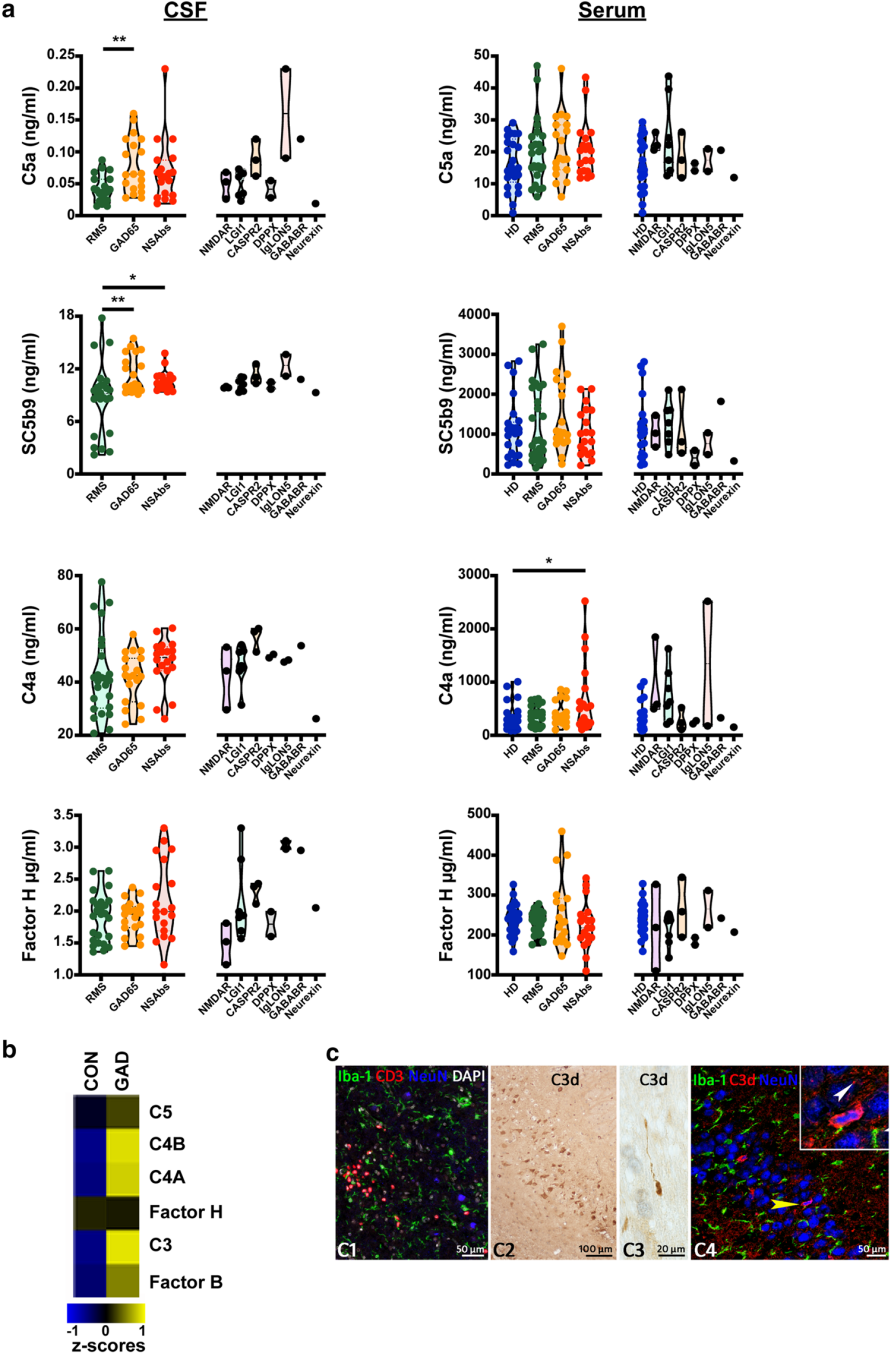
**a** Activated complement proteins in CSF and sera of NSAbs^+^ (*n* = 19) and GAD65-Ab^+^ (*n* = 19) encephalitis patients compared to RMS patients (*n* = 25) and HD (*n* = 25). Right panels depict levels in individual clinical entities of NSAbs-AE. *p ≤ 0.05, **p ≤ 0.001. **b** Microarray analysis of control (*n* = 7, CON) and GAD-Ab^+^ limbic encephalitis (*n* = 5, GAD) hippocampal tissue shows that mRNA expression for complement factors C3, C4A and C4B are strongly upregulated in GAD-Ab^+^ encephalitis. Shown are means of *z*-scores. (**c1**) Staining for T cells (CD3), Microglia (Iba-1) and neurons (NeuN) shows infiltration of T cells in the hippocampal parenchyma of a GAD-AE patient. DAPI is used as a nuclear counterstain. (**c2**) Immunohistochemistry for C3d shows hippocampal neurons. (**c3**) C3d reactivity also is seen in axonal spheroids in white matter tracts. (**c4**) Confocal fluorescence staining here shows NeuN^+^ neurons of the DG. The yellow arrowhead points toward a neuron with a condensed nucleus as enlarged in the inset. C3d staining is a representative image of one of the five patients that showed C3d reactivity

Deposition of activated complement proteins has previously been observed in biopsy material from patients with CASPR2-AE [5] and LGI1-AE [4]. NMDAR-specific Abs from patients with AE are able to bind complement in vitro, while deposits of complement were not detected in patients' brains [6]. To investigate whether complement activation is detectable within the CNS in GAD-Ab^+^ AE, we next analyzed hippocampi from patients with GAD-Ab^+^ limbic encephalitis (biopsies from resective epilepsy surgeries; all females; age, disease duration (mean ± SD, range): 52 ± 40, 1.2–291.6 months) for complement factor gene and protein expression. Compared to 7 control patients, who underwent temporal lobe epilepsy surgery for extra-hippocampal low-stage tumors, patients with GAD-Ab^+^ encephalitis showed substantially increased transcriptional levels for genes encoding complement proteins, such as C3, C4A and C4B (Fig. 1b). We next assessed complement activation in situ on a protein level. As negative controls, we used aged-matched autopsy material from non-neurological disease specimens while staining of plaques in cortex from Alzheimer´s disease patients was used as positive controls. C3d deposition could be visualized in 5 from 7 patients. Besides staining of serum in and around blood vessels, C3d immunoreactivity was detected in single or groups of neurons in various hippocampal regions, such as cornu ammonis (CA)1, CA4 and dentate gyrus and in axonal spheroids in white matter tracts. Some of these neurons showed signs of damage reflected by shrinkage of nuclei (Fig. 1c).

Our study demonstrates that CSF and neural cell-associated complement activation contribute to GAD Ab-associated AE and is not restricted to AE associated with NSAbs. Complement activation can result in direct cytotoxicity via formation of the membrane attack complex while additionally regulating multiple pathways from adhesion to inflammatory signaling or phagocytosis. As seen in chronic MS, and suggested here by staining of dystrophic axons and degenerating neurons, complement activation may represent a mechanism to remove neural structures in GAD Ab-associated AE [7].

To our knowledge, this is the first report on complement activation in the CSF and serum compartments in larger cohorts of patients with a broad spectrum of AE entities. Our data indicate that, in addition to cell-mediated immunity, complement deposition contributes to the pathology of GAD Ab-associated AE. The size of cohorts and the number of patients diagnosed with NSAb-AE entities, due to the rarity of AE, are a limitation of our report and requires validation and mechanistic analyses in larger cohorts.

## Supplementary Information

Below is the link to the electronic supplementary material.

Supplementary file1 (DOCX 19 KB)
