# Supplementary material for: Complement activation contributes to GAD antibody-associated encephalitis
Source: Acta Neuropathol. 2022 Jun 13;144(2):381–3. doi: 10.1007/s00401-022-02448-x (PMC9288364; doi:10.1007/s00401-022-02448-x)
Supplement: Supplementary file 1 — Supplementary file1 (DOCX 19 KB) [file 401_2022_2448_MOESM1_ESM.docx]

**ONLINE RESOURCE**

***Springer, Acta Neuropathologica***

**Complement activation contributes to GAD antibody-associated encephalitis**

# Omar Chuquisana ^1^, Christine Strippel ^1^, Anna M. Tröscher ^2,3^, Tobias Baumgartner ^4^, Attila Rácz ^4^, Christian W. Keller ^1^, Christian E. Elger ^4^, Nico Melzer ^5^, Stjepana Kovac ^1^, Heinz Wiendl ^1^, Jan Bauer ^2*^, Jan D. Lünemann ^1*^

^1^ Department of Neurology with Institute of Translational Neurology, University Hospital Münster, Münster 48149, Germany.

^2^ Department of Neuroimmunology, Center for Brain Research, Medical University of Vienna, Vienna, Austria.

^3^ Department of Neurology I, Neuromed Campus, Kepler University Hospital Linz, Austria

^4^ Department of Epileptology, University Hospital Bonn, Bonn, Germany.

^5^ Department of Neurology, Medical Faculty, Heinrich-Heine-University Düsseldorf, Düsseldorf, Germany.

^*^ Equally contributing senior authors

***Corresponding author:** Jan D. Lünemann, Department of Neurology with Institute of Translational Neurology, University Hospital Münster, Münster 48149, Germany: Phone: +49-251-83-53080; Fax: +49-25183-48199; Email: [jan.luenemann@ukmuenster.de](mailto:jan.luenemann@ukmuenster.de)

**Supplementary material**

**Supplementary Table 1, online resource.** Demographical and clinical characteristics of patients and healthy individuals assessed for complement protein CSF and serum levels.

|  | **AE** | | **RMS** | **HD** |
| --- | --- | --- | --- | --- |
| **N** | 38 | | 25 | 25 |
| **Age [mean, median, SD, (range)]** | 49, 50, 15.7 (22-81) | | 39, 46, 15.2 (15-71) | 50, 52, 10.5 (21-65) |
| **Sex (N, % female)** | 20 (52.6%) | | 13 (52%) | 12 (48%) |
|  | **Ab specific for surface Ag** | **Ab specific for GAD65 Ag** |  |  |
| **N** | 19 | 19 | 25 | 25 |
| **Age [mean, median, SD, (range)]** | 41, 57, 16 (22-81) | 41, 42, 11 (24-61) | 39, 46, 15.2 (15-71) | 50, 52, 10.5 (21-65) |
| **Sex (N, % female)** | 42.1% | 63.2% | 13 (52%) | 12 (48%) |
| **Ab specificity** | LGI1: 7 (37%)  CASPR2: 3 (16%)  NMDAR: 3 (16%)  IgLON 5: 2 (10.5%)  DPPX: 2 (10.5%)  Neurexin: 1 (5%)  GABABR: 1 (5%) | GAD65-LE: 18 (95%)  GAD65-SPS: 1 (5%) | n.a. | n.a. |
| **Disease duration, months [mean, median, SD, (range)]** | 12, 2, 27 (0.11-120) | 79, 72, 55 (3-204) | 28, 1, 100 (0.10-480) | n.a. |
| **Immunotherapy at time of blood draw (%)** | 0/19 (0%) | 0/19 (0%)* | 1/25 | n.a. |
| **Inflammatory Changes in the MRI** | 12/19 (63%) | 12/19 (63%) | 25/25 (100%) | n.a. |
| **Routine CSF Parameters** |  |  |  |  |
| 1. **Cells/µl, median (range)** | 3 (0-30) | 1 (0-6) | 2 (0-72) | n.a. |
| 1. **Pleocytosis (cells > 4/µl), N (%)** | 8/19 (42%) | 1/19 (5.3%) | 10/25 (40%) | n.a. |
| 1. **Blood-CSF barrier dysfunction, N (%)** | 6/19 (32%) | 1/19 (5.3%) | 11/25 (44%) | n.a. |
| 1. **IgG-Intrathecal immunoglobulin synthesis, N (%)** | 1/19 (5.5%) | 1/19 (5.3%) | 11/25 (44%) | n.a. |
| 1. **IgM-intrathecal immunoglobulin synthesis, N (%)** | 2/19 (11%) | 0/19 (0%) | 1/25 (4%) | n.a. |

Ab=antibody; AE=autoimmune encephalitis; Ag=antigen; CASPR2=contactin-associated protein-like 2; CSF=cerebrospinal fluid; DPPX**=**dipeptidyl-peptidase-like protein-6; GABABR=gamma-aminobutyric acid b receptor; GAD65=glutamic acid decarboxylase 65-kilodalton protein; HD=healthy donor; Ig=immunoglobulin; IgLON 5=immunoglobulin-like cell adhesion molecule 5; LE=limbic encephalitis; LGI1=leucine-rich glioma inactivated 1; n.a.=not applicable; NMDAR=N-methyl-D-aspartate receptor. SPS=stiff-person-syndrome. * Treatment information regarding immunotherapy at the time of blood draw was available for 15 out of 19 GAD Ab^+^ patients. Immunosuppressive therapies included intravenous methylprednisolone (N=13), in 6 cases combined with immunoadsorption (N=5) or plasma exchange (N=1). Long term immunotherapy included Azathioprin (N=2), MMF (N=1), Rituximab (N=3), Basiliximab (combined with IV steroids, N=2) and cyclophosphamide (N=1). Follow up visits after 12 months or longer were available for all of these patients, of whom five showed a beneficial response to immunotherapy (reduction in seizures or improvement of modified Rankin Score) as perceived by the patient and the treating physician

**Material and methods.**

**Complement quantification, gene expression and immunohistochemistry**

Concentrations of C5a, chemoattractant cleavage product of complement factor 5, the soluble terminal complement complex sC5b9, the cleavage product C4a, specific for the activation of the classical pathway, and the complement inhibitory protein factor H were quantified simultaneously in cerebrospinal fluid (CSF) and serum specimens using a multiplex ELISA based on chemiluminescence (Quidel, San Diego, USA, cat. number: A900). Data was obtained with Imager L from Quansys, using Q-View Software 3.11 for analysis. The non-parametric Kruskal-Wallis test was performed to compare levels of complement proteins between clinical cohorts. The study was approved by the Ethics Committee of the of the University of Münster (2015-088-f-S; 2019-712-f-S). All subjects provided written informed consent prior to participation. Two ng RNA per sample were hybridised to a GeneChip™ Human Gene 2.1 ST 16-Array Plate (Affymetrix, Thermo Fisher Scientific, Cat. Nr.: 902136) and scanned with GeneTitanTM MC Instrument (Affymetrix, Thermo Fisher Scientific) at the Genomic Core Facility of the Medical University of Vienna. RMA-sketch normalized gene signals were extracted from the TAC software (version 4.0) and means of z-scores were calculated for the genes of interest. The heatmap was created using the MeV software (version 4.8.1). Immunohistochemistry and confocal microscopy was performed for CD3 (Neomarkers #RM9107-S) for T cells, C3d (DakoCytomation A0063) together with NeuN (Chemicon, A60) for neurons and Iba-1 (Wako #019-19741) for microglia.
